# Predictors for improvement of xerostomia in nasopharyngeal carcinoma patients receiving intensity-modulated radiotherapy

**DOI:** 10.1097/MD.0000000000017030

**Published:** 2019-09-06

**Authors:** Xin-Bin Pan, Yang Liu, Shi-Ting Huang, Kai-Hua Chen, Yan-Ming Jiang, Xiao-Dong Zhu

**Affiliations:** Department of Radiation Oncology, Cancer Hospital of Guangxi Medical University, Nanning, Guangxi, PR China.

**Keywords:** intensity-modulated radiotherapy, nasopharyngeal carcinoma, xerostomia

## Abstract

To identify predictors for improvement of xerostomia in patients with nasopharyngeal carcinoma (NPC) treated with intensity-modulated radiotherapy (IMRT).

Patients diagnosed with stage I-IVb NPC (according to the 7th edition of the American Joint Committee on Cancer) between September 2015 and March 2016 were retrospectively analyzed. All the patients received IMRT. Predictors for improvement of xerostomia were analyzed using logistic regression analysis. Receiver operating characteristic curve analysis was used to identify the most appropriate cut-off values for predicting factors.

This study included 195 patients: xerostomia improved in 109 patients and xerostomia remained unchanged in 86 patients. Volume of the parotid gland ≤52.2 cm^3^ was a risk factor for xerostomia improvement (odds ratio [OR] = 3.506, 95% confidence interval [CI]: 1.932–6.362, *P* = .001). The mean dose of <39 Gy to the ipsilateral parotid gland was a protective factor (OR = 0.417, 95% CI: 0.271–0.641, *P* = .001). V30 of the contralateral parotid gland ≤52% was a protective factor (OR = 0.593, 95% CI: 0.462–0.760, *P* = .001).

Volume of the parotid gland, the mean dose of the ipsilateral parotid gland, and V30 of the contralateral parotid gland were independent predictors for improvement of xerostomia.

## 1 Introduction

Nasopharyngeal carcinoma (NPC) is a highly endemic cancer in Southern China.^[1,2]^ Radiotherapy with or without chemotherapy is the primary treatment modality for NPC. Xerostomia is a common complication after radiotherapy.^[3]^ Xerostomia affects the quality of life, including speech, nutrition, taste, sleep, and communication.^[4,5]^ Thus, reducing xerostomia would improve the quality of life of patients who have a high survival rate.^[6–8]^

Xerostomia and sticky saliva were common problems observed 2 months after treatment.^[9]^ Thereafter, continuous improvement occurred. McMillan et al^[9]^ reported that salivary flow recovered to at least 25% of baseline in most patients after 12 months. However, Kwong et al^[10]^ found that only 60% of patients recovered at least 25% of their baseline saliva secretion. Moreover, many patients did not recover saliva secretion in the clinical settings, with xerostomia being consistent over time.^[11]^

The mean dose to the parotid gland was the most important factor that influenced the parotid function.^[12]^ However, the most appropriate cut-off points for the mean dose differed significantly.^[12–16]^ Moreover, the potential predictive factors of xerostomia recovery are still unclear, including the volume of the parotid glands, age, and chemotherapy. Therefore, the current study was conducted to assess the predictive factors for the improvement of xerostomia in patients with NPC treated with intensity-modulated radiotherapy (IMRT).

## 2 Methods

### 2.1 Patients

This retrospective cohort study was conducted at the Affiliated Tumor Hospital of the Guangxi Medical University. Patients who were diagnosed with stage I-IVb NPC (according to the 7th edition of the American Joint Committee on Cancer) between September 2015 and March 2016 were included. All patients received IMRT with or without chemotherapy.

This study was approved by the Affiliated Tumor Hospital of Guangxi Medical University Ethics Committee. But, informed consent was not available due to the retrospective nature.

### 2.2 Treatment

Radiotherapy was performed as described previously.^[11]^ Patients received IMRT per the International Commission on Radiation Units and Measurements Report 62 guidelines. The gross tumor volume of the nasopharynx (GTVnx) and gross tumor volume of the cervical lymph nodes (GTVnd) were quantified by using a computed tomography (CT) or magnetic resonance imaging (MRI) scans. The high-risk clinical target volume (CTV1) included the GTVnx plus a 5 to 10-mm margin to encompass the high-risk sites of microscopic extension and the whole nasopharynx. The low-risk clinical target volume (CTV2) was defined as the CTV1 plus a 5 to 10-mm margin to encompass the low-risk sites of microscopic extension, including the skull base, clivus, sphenoid sinus, parapharyngeal space, pterygoid fossae, posterior parts of the nasal cavity, pterygopalatine fossae, retropharyngeal nodal regions, and the elective neck area from level IB to V. The planning target volume (PTV) was defined by adding a 3-mm margin to the GTV or CTV. The prescribed radiation doses were 70.06 to 72.32 Gy for the PGTVnx, 66.00 to 72.32 Gy for the PGTVnd, 60.00 to 62.00 Gy for the PCTV1, and 54.00 to 55.80 Gy for the PCTV2.

Induction chemotherapy included 60 mg/m^2^ of docetaxel for 1 day, 60 mg/m^2^ of cisplatin for 1 day, and 600 mg/m^2^/day of 5-fluorouracil as a continuous intravenous infusion for 120 hours for 3 cycles. Concurrent chemotherapy was 100 mg/m^2^ of cisplatin for 1 or 3 days with 1 cycle on days 1, 22, and 43 during radiotherapy.

### 2.3 Dosimetric parameters of the parotid glands

All the parotid glands were contoured by a single physician (L.Y) based on the fusion images of MRI-CT-Sim to exclude observer variability on contouring the parotid glands. No margin was added during treatment planning for the parotid glands. The dose-volume histograms were calculated by using Pinnacle^3^ 9.8 (Philips Co., Eindhoven, Netherlands). The initial volume of the parotid glands, mean dose, and V30 of the ipsilateral and contralateral parotid glands were calculated.

### 2.4 Xerostomia assessment

Patients were followed-up every 3 months during the first 2 years, every 6 months for the next 3 years, and then annually thereafter. Xerostomia was assessed at 3 months, 6 months, and 12 months after treatment according to the Radiation Therapy Oncology Group/European Organization for Research and Treatment of Cancer (RTOG/EORTC) system.^[17]^ Slight dryness not affecting the quality of life correlated with grade 1 toxicity. Moderate dryness that required a water bottle was considered grade 2 toxicity. Severe dryness that caused a profound change in the quality of life was considered grade 3 toxicity.

Patients were divided into the xerostomia improved and xerostomia unchanged groups. Xerostomia was considered to have improved when the xerostomia score recovered by at least 1 grade during follow-up. Xerostomia was considered to be unchanged when the xerostomia score was constant.

### 2.5 Statistical analysis

Continuous variables were analyzed using the Student *t* test. Categorical variables were analyzed by using the *χ*^2^ test. Predictors for improving xerostomia were analyzed using logistic regression analysis. The receiver operating characteristic (ROC) curve analysis was used to assess the most appropriate cut-off points for potential predictors. Statistical analyses were performed using SPSS Statistics Version 23.0 software (IBM Co., Armonk, NY). Two-tailed *P* < .05 was considered statistically significant.

## 3 Results

### 3.1 Patient characteristics

This study included 195 patients: 109 patients in the xerostomia improved group and 86 patients in the xerostomia unchanged group. The patient characteristics are shown in Table 1. All the patients were followed-up for >12 months.

**Table 1:**
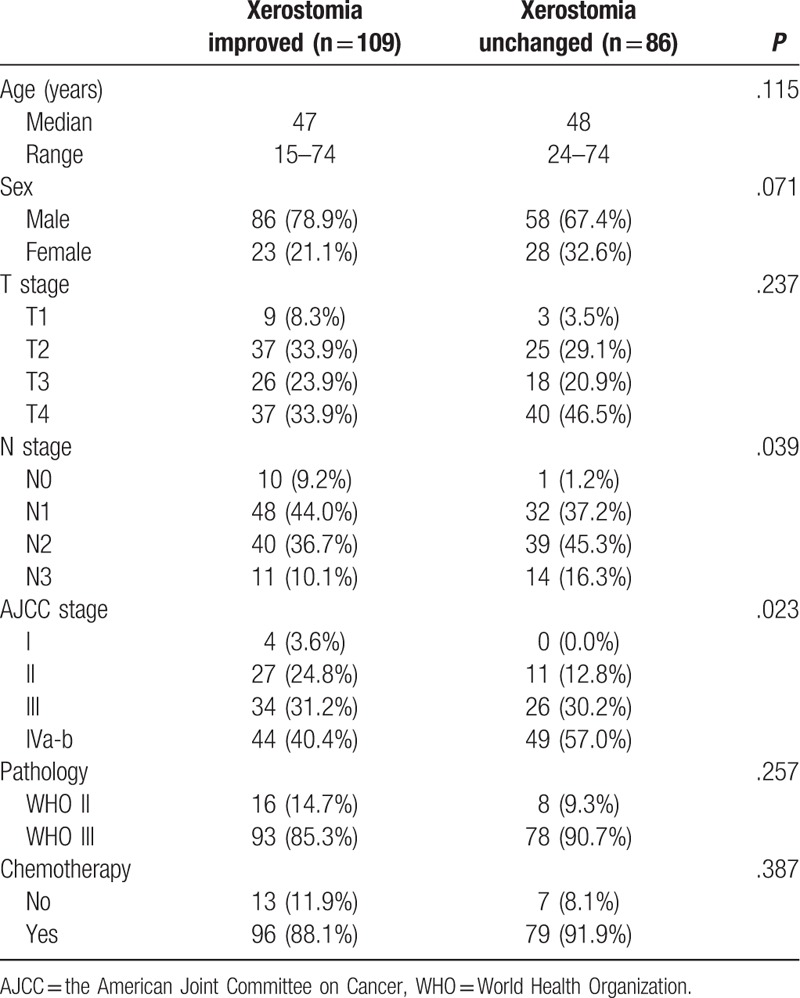
Patient characteristics.

### 3.2 Predictors for improvement of xerostomia

The N stage, the volume of the parotid glands, mean dose of the ipsilateral parotid gland, mean dose of the contralateral parotid gland, V30 of the ipsilateral parotid gland, and V30 of the contralateral parotid gland were predictive factors for the improvement of xerostomia on univariate analysis (Table 2). On multivariate logistic regression analysis, the volume of the parotid gland (*P* = .008), mean dose of the ipsilateral parotid gland (*P* = .014), and V30 of the contralateral parotid gland (*P* = .008) were independent predictors (Table 2).

**Table 2:**
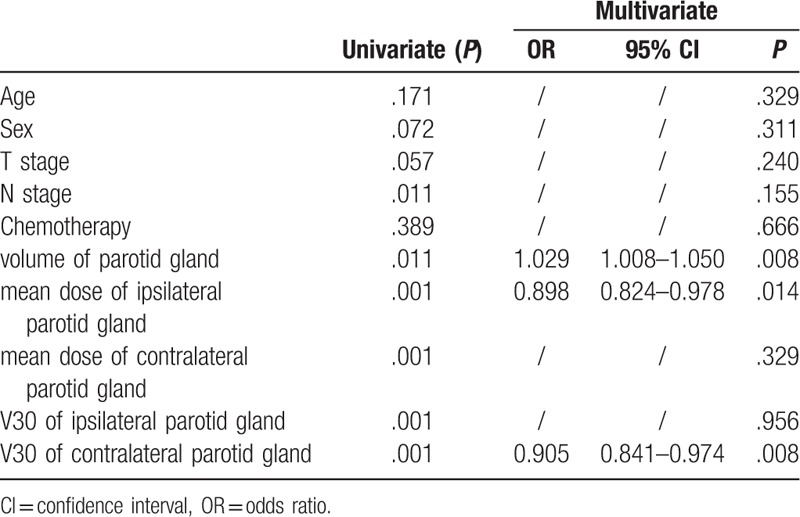
Univariate and multivariate analysis of predictors for xerostomia improved.

**Figure 1:**
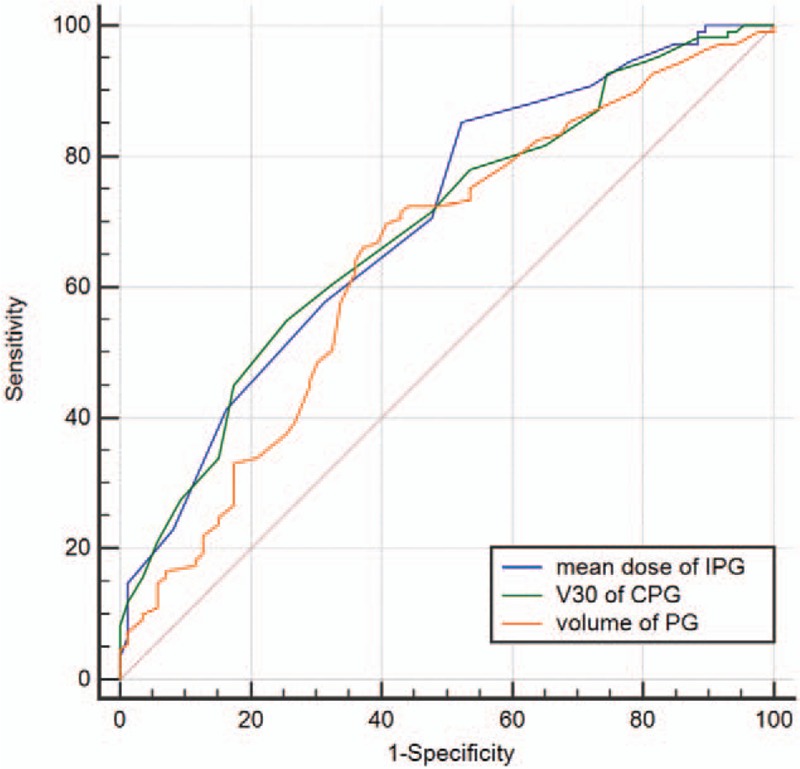
Predictive ability of predictors for xerostomia improved in receiver operating characteristic curve analysis. IPG: ipsilateral parotid gland, CPG: contralateral parotid gland, PB: parotid gland.

The cut-off values determined by using the ROC curve were 52.2 cm^3^, 39 Gy, and 52% for the volume of the parotid gland, mean dose of the ipsilateral parotid gland, and V30 of the contralateral parotid gland, respectively. The area under the curve (AUC) was 0.645, 0.700, and 0.689, respectively (Fig. 1).

Table 3 shows the improvement of xerostomia according to the predictive factors. The volume of the parotid gland ≤52.2 cm^3^ was a risk factor for xerostomia improvement compared to the volume >52.2 cm^3^ (odds ratio [OR] = 3.506, 95% confidence interval [CI]: 1.932–6.362, *P* = .001). The mean dose of the ipsilateral parotid gland <39 Gy was a protective factor (OR = 0.417, 95% CI: 0.271–0.641, *P* = .001). V30 of the contralateral parotid gland ≤52% was a protective factor (OR = 0.593, 95% CI: 0.462–0.760, *P* = .001).

**Table 3:**
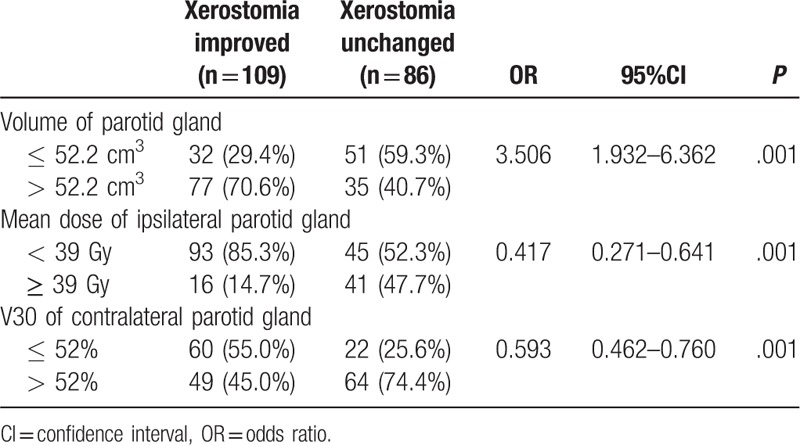
Improvement of xerostomia according to predict factors.

## 4 Discussion

The results of the current study indicated that a larger volume of the parotid gland, lower mean dose of the ipsilateral parotid gland, and lower V30 of the contralateral parotid gland were protective factors for xerostomia improvement. Accordingly, clinicians should aim to achieve these objectives to the maximum possible extent to reduce irradiation-induced xerostomia.

The initial volume of the parotid glands was correlated with the grade of xerostomia. Nishimura et al^[18]^ reported that patients with smaller parotid glands (*≤*38.8 cm^3^) experienced grade 2 to 3 xerostomia more often compared to patients with larger parotid glands (*P* < .05). Compared to a smaller volume of the parotid glands, a larger volume results in a lower mean dose, which causes the loss of fewer acinar cells. Thus, salivary function can be partially preserved and gradually improves over time.^[9,10,19,20]^ The current study revealed that the cut-off value for the volume of the parotid gland was ≤52.2 cm^3^. The difference between the cut-off values may have resulted from the different sample sizes and the types of included patients in the 2 studies.

In clinical practice, a mean dose of <26 Gy to at least 1 parotid gland was hard to achieve. A V30 of <50% of the parotid gland was a commonly used criterion. However, this criterion usually cannot be achieved, especially for locoregionally advanced NPC. The V30 of the ipsilateral parotid gland is assumed to be 60% or higher. We usually reduced the doses of the contralateral parotid gland to the maximum possible extent. In the current study, V30 of the contralateral parotid gland ≤52% was a predictive factor of improvement of xerostomia. It is possible that V30 of the contralateral parotid gland ≤52% resulted in the preservation of 50% of acinar cells after 30 Gy irradiation.^[21]^ Thus, the parotid gland function may recover continuously.

The mean dose of <26 Gy for at least 1 parotid gland should be a planning goal.^[13,14]^ This dose was sufficient to achieve complete recovery of pre-treatment salivary flow rates. However, the current study results showed that the cut-off value for the mean dose of the ipsilateral parotid gland was 39 Gy. This cut-off value was much higher than that reported in previous studies. The difference in the cut-off values may be because previous studies included head and neck cancers, but not NPC; the parotid glands receive higher doses in patients with NPC compared to those with laryngeal cancer or hypopharyngeal carcinoma. In addition, the current study used physician-assessed scores as endpoints, while previous studies used salivary flow measurement. However, the correlations between the average RTOG/EORTC grades and the salivary flow rates were not significant.^[22]^

In the present study, multivariate logistic regression analysis showed that the volume of the parotid gland, mean dose of the ipsilateral parotid gland, and V30 of the contralateral parotid gland were independent predictive factors. However, the 3 predictors may have potential correlations. Hey et al^[13]^ found that the radiation volume, which depends on tumor site, did significantly influence the mean dose of the parotid glands, and thus on the saliva flow and recovery of the parotid glands. Nevertheless, further studies are needed to determine the potential correlations among these predictors.

This study had some limitations. The objective parotid function as measured by salivary flow is more accurate, with most previous studies using salivary flow as the main measurement.^[9,10,13–16,20]^ However, the improvement in objective parotid function as measured by salivary flow is not always accompanied with improved patient-reported xerostomia.^[23]^ Moreover, patient self-reported, rather than physician-assessed, scores should be the main end-point for evaluating xerostomia.^[22]^ However, we only assessed xerostomia according to the RTOG/EORTC system.^[17]^ Therefore, our results should be verified in a prospective cohort study with salivary flow measurement by using a patient self-reported validated xerostomia questionnaire.

In conclusion, the volume of the parotid gland, mean dose of the ipsilateral parotid gland, and V30 of the contralateral parotid gland were predictors for xerostomia improvement of patients with NPC treated with IMRT.

## Acknowledgments

We would like to thank Editage (www.editage.com) for English language editing.

## Author contributions

**Conceptualization:** Xin-Bin Pan, Xiao-Dong Zhu.

**Data curation:** Yang Liu, Yan-Ming Jiang.

**Formal analysis:** Yang Liu, Kai-Hua Chen.

**Methodology:** Xin-Bin Pan, Yang Liu, Shi-Ting Huang.

**Writing – original draft:** Xin-Bin Pan.

**Writing – review & editing:** Xiao-Dong Zhu.
